# Design, characterization and in vitro evaluation of thin films enriched by tannic acid complexed by Fe(III) ions

**DOI:** 10.1007/s40204-020-00146-z

**Published:** 2020-11-21

**Authors:** B. Kaczmarek, O. Mazur, O. Miłek, M. Michalska-Sionkowska, A. Das, A. Jaiswal, J. Vishnu, K. Tiwari, A. Sionkowska, A. M. Osyczka, G. Manivasagam

**Affiliations:** 1 Department of Biomaterials and Cosmetics Chemistry, Faculty of Chemistry, Nicolaus Copernicus University in Toruń, Gagarin 7, 87-100 Torun, Poland; 2 Department of Cell Biology and Imaging, Institute of Zoology and Biomedical Research, Faculty of Biology, Jagiellonian University in Kraków, Gronostajowa 7, Krakow, Poland; 3 Department of Environmental Microbiology and Biotechnology, Faculty of Biology and Veterinary Science, Nicolaus Copernicus University in Toruń, Lwowska 1, Torun, Poland; 4 Centre for Biomaterials Cellular and Molecular Theranostics, Vellore Institute of Technology, Vellore, Tamil Nadu India

**Keywords:** Complexation, Iron (III) complexes, Polyphenol

## Abstract

Materials based on carbohydrate polymers may be used for biomedical application. However, materials based on natural polymers have weak physicochemical properties. Thereby, there is a challenge to improve their properties without initiation of toxicity. The alternative method compared to toxic chemical agents’ addition is the use of metal complexation method. In this study, chitosan/tannic acid mixtures modified by Fe(III) complexation are proposed and tested for potential applications as wound dressings. Thereby, surface properties, blood compatibility as well as platelet adhesion was tested. In addition, the periodontal ligament stromal cells compatibility studies were carried out. The results showed that the iron(III) addition to chitosan/tannic acid mixture improves properties due to a decrease in the surface free energy and exhibited a reduction in the hemolysis rate (below 5%). Moreover, cells cultured on the surface of films with Fe(III) showed higher metabolic activity. The current findings allow for the medical application of the proposed materials as wound dressings.

## Introduction

Materials for skin dressing and wound healing should be biocompatible, bioactive, provide defensive action against bacteria and stimulate angiogenesis to support the active healing process (Kaczmarek et al. 2019a). Conventional scaffold materials are made from materials used for such applications which possess the undesirable properties such as absorption of fluids from the wound, clinging to the wound surface, and causing injury during the removal of dressing (Singh et al. 2013). Novel dressing materials are non-adherent to the wound and have the ability to create a moist environment near the wound by keeping the wound fluids in contact with the wound (Singh et al. 2013; Phaechamud et al. 2016; Kaczmarek et al. 2019b).

Chitosan, a linear polymer of *α*-(1 → 4)-linked 2-amino-2-deoxy-*β*-d-glucopyranose (Dutta et al. 2004) is the key derivative of the second most abundant polysaccharide and is derived by the process of deacetylation from chitin. Although it is biocompatible and non-toxic, it does not possess the desired mechanical properties. In such a case, it is mixed with other polymers to improve their physicochemical properties (Schwarz et al. 2012; Sobahi et al. 2013).

Tannic acid is obtained from natural plants and has widespread applications in distinctive fields such as medicine (Chung et al. 1998), food (Martinez et al. 2006), tanning (Colak et al. 2010), cosmetic (Gülçin et al. 2010), and metallurgy. It is composed of repeating unit glucose and gallic acid, and contains multiple phenolic hydroxyl groups in its structure which provide it with distinguished physical, chemical, biological, and pharmacological properties (Slawinska et al. 1979; Mollenhauer and Morré 1987). Skin is always a favorable medium for microbes and hence there exist higher chances of wound infection. It has been proved earlier that tannic acid possesses the excellent antimicrobial properties. Inhibition zones were being observed with different concentrations of tannic acid and it was found that 3% tannic acid concentration was the most effective (Fu and Chen 2019).

The tannic acid structure consists of a central glucose molecule esterified at all five hydroxyls with two gallic acids. It shows an intense absorption band at 274 nm and acts as a UV-absorbing chromophore. Protonated phenolic groups cannot react with metal ions. De-protonated phenolic groups exposed to oxygen anions can react with metal ions to form a stable five-membered ring complex. Although the third hydroxyl group of gallic acid does not take part in the reaction with metal ions, it will assist in stabilizing the complex through the delocalization of the lone pairs in the other two hydroxyl groups. The metal complexation method may be an alternative cost-effective method to the costly cross-linking processes which are based on modifying the material properties by adding special cross-linking agents (Fu and Chen 2019).

In this study, novel polymeric blends of chitosan and tannic acid complexed with Fe(III) were obtained in the form of films. Previously, chitosan and tannic acid mixtures have been studied (Kaczmarek et al. 2019b). The complex formation of tannic acid by Fe(III) addition has been already reported (Fu and Chen 2019; Kim et al. 2015). However, its combination with chitosan is novel. Metal complexing modification may be an alternative approach to cross-link processes of chitosan mainly proposed to improve materials properties in an inexpensive and effective manner.

## Materials and methods

### Materials

Chitosan (CTS), tannic acid (TA), iron (III) sulfate, and phosphate-buffered saline (PBS) are commercial compounds purchased from the Sigma-Aldrich company (Poznan, Poland). The deacetylation degree (DD, %) of chitosan was 78%, and the molecular weight was 1.8 × 10^6^ g/mol. The molecular weight of tannic acid was 1701.2 g/mol.

### Sample preparation

**Fig. 1:**
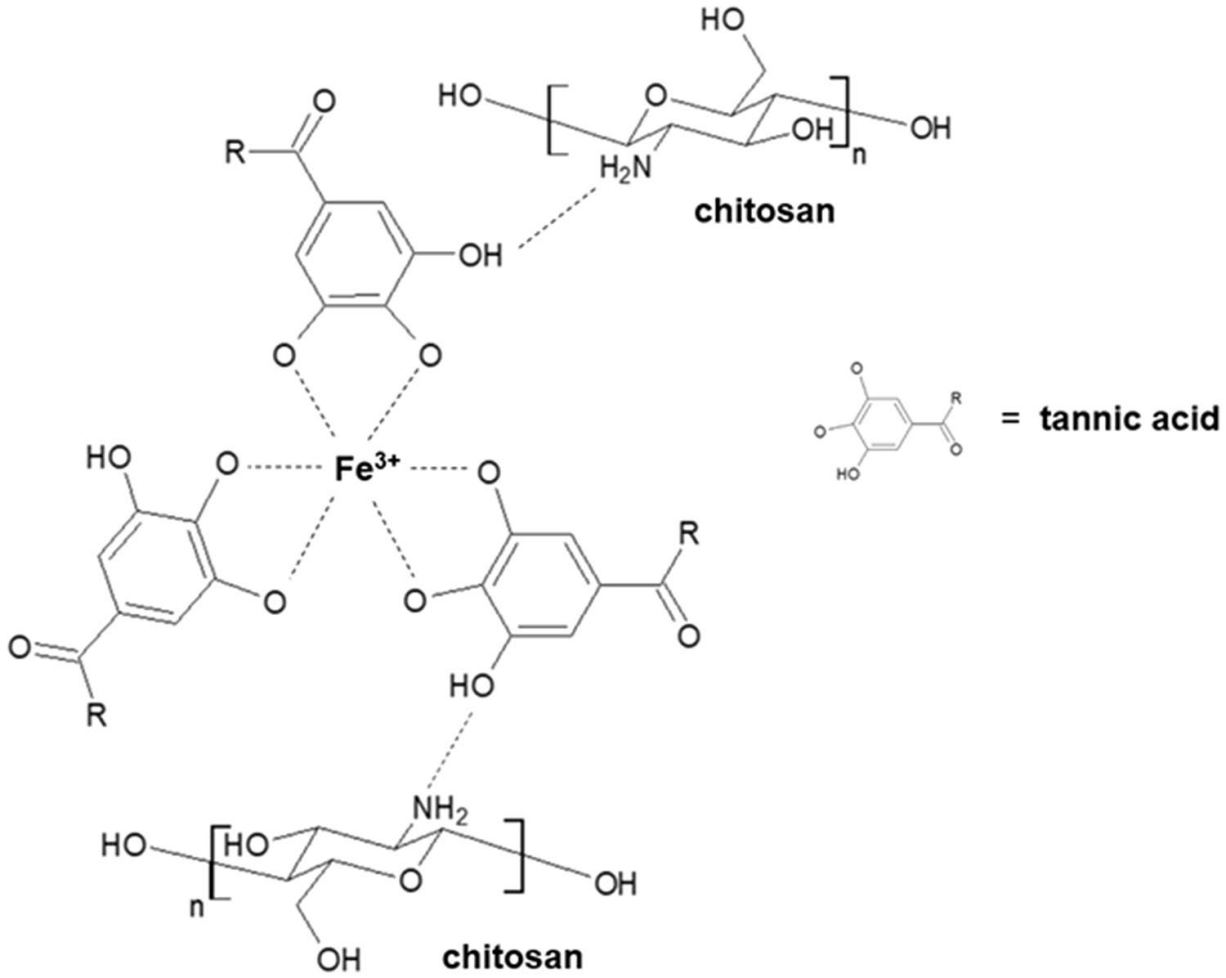
The schematic cross-linking process between chitosan, tannic acid and Fe(III) ions

The materials preparation method has been modified compared to the methodology published (Yang et al. 2019) where tannic acid has been oxidized by the use of laccase. In our study, chitosan and tannic acid were dissolved in 0.1 M acetic acid, separately, at a concentration of 2%. The mixtures of chitosan and tannic acid were prepared in the weight ratios of 80/20, 50/50, 20/80 (Kaczmarek et al. 2019b). All the mixtures were obtained by mixing chitosan and tannic acid solutions on a magnetic stirrer. The addition of iron ions was proposed in different ratios. Iron (III) solution was added to CTS/TA mixtures as 1, 5 and 10% additive based on the chitosan and tannic acid content. As a result, we obtained materials with different molar ratios between tannic acid and Fe(III). Such prepared chitosan and TA-Fe(III) complexes were placed in the plastic holder for 48 h for the solvent evaporation. The schematic illustration of the cross-linking process is presented in Fig. 1.

### Fourier transform infrared spectroscopy–attenuated total reflectance (FTIR–ATR)

FTIR–ATR spectra were obtained for each type of film in the range 1800–800 cm^−1^ by the Nicolet spectrometer (iS110) equipped with diamond crystal with the resolution 4 cm^−1^. Spectra were taken with 32 scans.

### Contact angle measurement

The most frequently applied approach for quantifying the wettability of materials with a smooth surface is the sessile drop method, whereby, the contact angle is measured by the liquid drop from the placement (Kaczmarek et al. 2019a). Surface free energy (γs), its polar (γsP) and dispersive (γsD) components can be calculated by contact angle measurement, in which non-covalent forces between the liquid and film surface are formed by Owens–Wendt method, which enables the dispersive and polar components of surface free energy to be obtained. The contact angles of two liquids: water and diiodomethane were measured at a constant temperature using a goniometer equipped with a system of drop shape analysis (DS10 Control Unit, Krüss, Germany).

### Maximum tensile strength

The maximum tensile strength was determined during the measurement with the use of testing machine Z.05 (Zwick/Roell, Germany) with the parameters as initial force 0.1 MPa and the velocity of 5 mm/min. Samples were prepared by the cutting of the defined shape of a paddle. Measurement was carried out at room conditions (*n* = 5). Results are presented as a mean value with standard deviation.

### Blood compatibility

Anti-coagulated sheep blood (0.2 mL) was added to 10 mL of physiological saline solution containing different specimens (1 cm^2^ area). Positive and negative samples were prepared by adding 0.2 mL of fresh blood to water ([OD] positive) and physiological saline ([OD] negative), respectively. All the test tubes were incubated at 37 °C. After 1 h, the suspension was centrifuged at 1000 rpm for 10 min and absorbance of the supernatant of each tube was measured by microplate reader Multiscan FC (Thermo Fisher Scientific, Waltham, USA) at 540 nm. Each sample was prepared in triplicate. Hemolysis rate was calculated using the equation:

1 $$\mathrm{rate\, of \,hemolysis}\, \left[\%\right]=\frac{\left[\mathrm{OD}\right]\mathrm{specimen} -\left[\mathrm{OD}\right]\mathrm{negative}}{\left[\mathrm{OD}\right]\mathrm{positive}-\left[\mathrm{OD}\right]\mathrm{negative}}*100\%.$$

### Platelet adhesion tests

Platelet-rich plasma (PRP) was obtained by centrifuging the whole blood at 2500 rpm for 10 min. The samples were sterilized using absolute ethanol followed by drying for 5 min. Samples with dimensions 7 mm × 7 mm × 2 mm were placed in 12-well plates. The samples were equilibrated for 6 h in 3 mL phosphate-buffered saline (PBS) at room temperature. PBS was replaced with 3 mL of PRP and samples were incubated for 90 min at 37 °C. Subsequently, PRP was removed and samples were washed thrice with 3 mL of PBS. The cells were fixed using 2.5% glutaraldehyde for 30 min. After fixing, the samples were washed with PBS and subsequently dehydrated using gradient washing in ethanol–water solutions. The samples were imaged using a Scanning Electron Microscope (SEM) (LEO Electron Microscopy Ltd, England).

### Materials preparation for cell culture studies

The materials for cell culture studies were prepared in the form of thin foils. The foils were soaked in 70% EtOH and rinsed with sterile PBS (BioShop) to wash out alcohol residue. Then, the materials were placed at the bottom of the 24-well plates (Nest) and pushed to the bottom with sterile rings made out of medical polypropylene. Later, the foils were sterilized under UV light with the laminar flow for 30 min. The materials made out of 50CTS/50TA with the addition of 10% Fe(III) were very fragile and were not tested in cell culture.

### Establishing cell cultures on the experimental films

Stromal cells used for this study were harvested from periodontal ligament of 31-year-old female, according to the protocol described (Bakkar et al. 2017) (Institutional Review Board protocol nr 1072.6120.253.2017). PDLSC (periodontal ligament stromal cells) were seeded directly onto material foils or tissue culture plastic as a control (TCP, Nest) at a density of 2 × 10^4^/cm^2^ in 1 mL of medium (84% Alpha-MEM Gibco, 15% FBS Gibco, 1% ZellShield Minerva Biolabs). The medium was exchanged on day 2. MTS assay (CellTiter 96® AQueous One Solution Cell Proliferation Assay, Promega) was performed on day 6 to determine the viability of cells, based on their metabolic activity. Prior to MTS assay, cells on materials were rinsed with PBS and then supplemented with 200 μL of phenol-free Alpha-MEM with 10 × diluted MTS reagent. The reactions were developed in CO_2_ incubator until the visible change of color in comparison to a blank (phenol-free Alpha-MEM with 10 × diluted MTS reagent in well without cells). Then, the products of the reactions were transferred to individual wells in 96-well plates (Falcon) and absorbance was measured at 492 nm using a microplate reader (SpectraMax iD3 Molecular Devices). The intensity of the developed product was directly proportional to the amount of metabolically active cells, according to the technical bulletin of CellTiter 96® AQueous One Solution Cell Proliferation Assay, Promega.

### Statistical analysis

All MTS tests were performed in triplicates or quadruplicates. Absorbance of blank was subtracted from each of MTS absorbance values. Then, MTS absorbance values were averaged (mean value). Results were statistically analyzed with one-way ANOVA and post hoc Tukey with *p* < 0.05 considered significant.

## Results and discussion

### Fourier transform infrared spectroscopy–attenuated total reflectance (FTIR–ATR)

**Fig. 2:**
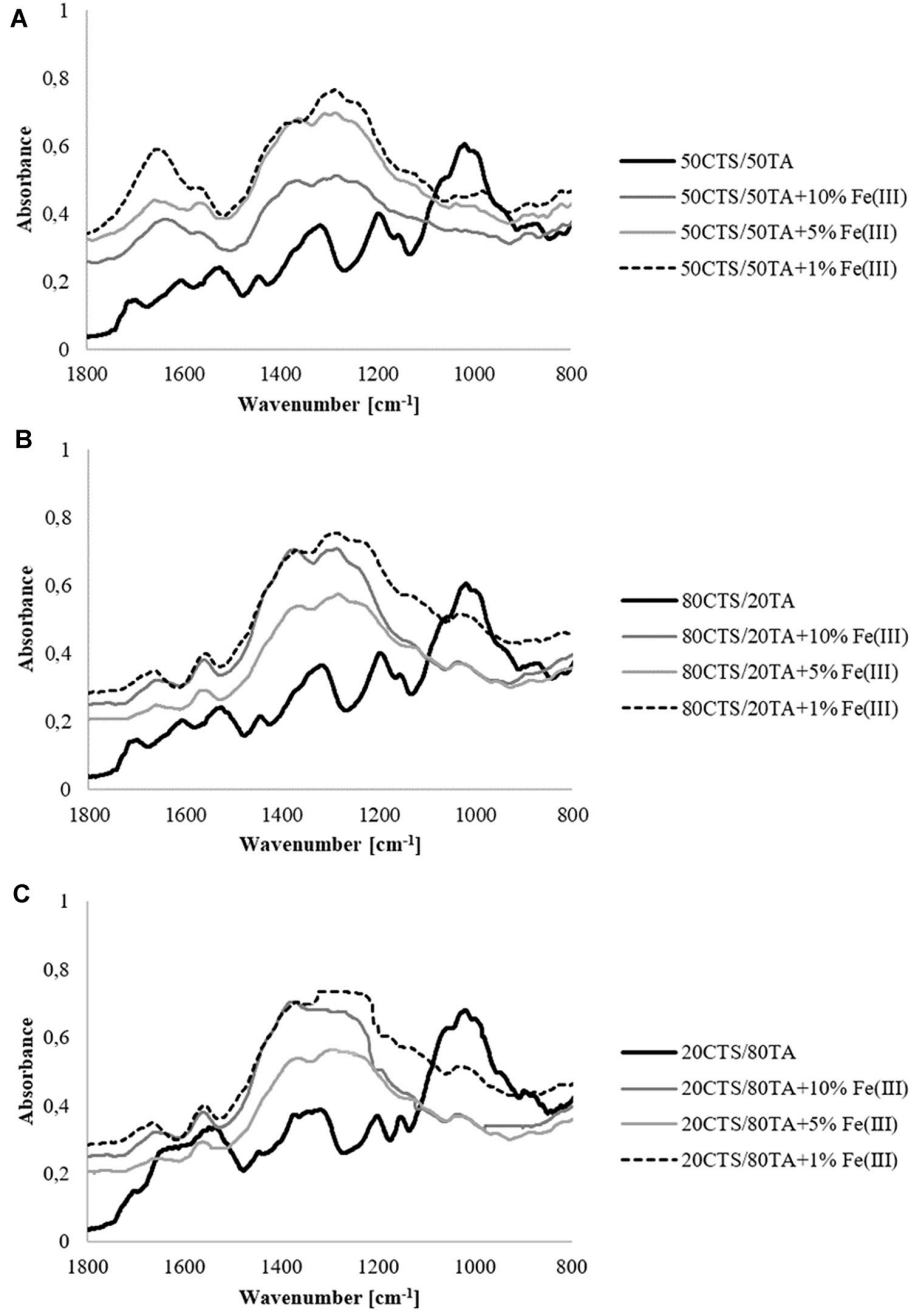
The FTIR–ATR spectra of **a** 50CTS/50TA, **b** 80CTS/20TA, **c** 20CTS/80TA, modified by 10, 5 and 1% addition of Fe(III)

The FTIR–ATR spectra (Fig. 2) allow determining the presence of functional groups of the materials’ components as well as distinct changes in the chemical interactions. The peak around 1064 cm^−1^ which represents the stretch motion of C–O–C is present in the spectra of chitosan/tannic acid in all three tested ratios. However, it is absent in the spectra of materials with Fe(III) addition. It is the result of the cross-linking process which occurs between material components. Moreover, it was observed that films with Fe(III) addition showed higher peaks in the range of 1400–1200 cm^−1^ compared to the materials without iron. In this rang, the peaks from the C–N and C–O bands may be distinguished. It suggests that the presence of Fe(III) reorganizes the interactions between chitosan and tannic acid.

### Contact angle measurement

Contact angle measurement is carried out to consider the material surface integration with surround environment. The surface free energy is energy resulting from the “dangling bonds” exposed at material's surface. The results of the contact angle measurement and surface free energy calculation are shown in Table 1.

**Table 1:** The surface free energy (γs), its polar (γsP) and dispersive (γsD) components of films based on chitosan and tannic acid–Fe(III) ratios

Specimen	γs [mJ/m^2^]	γsP [mJ/m^2^]	γsD [mJ/m^2^]
50CTS/50TA	38.20 ± 0.36	6.84 ± 0.15	23.93 ± 0.21
50CTS/50TA + 10% Fe(III)	34.83 ± 0.47	9.13 ± 0.15	25.70 ± 0.31
50CTS/50TA + 5% Fe(III)	30.29 ± 0.91	7.00 ± 0.56	23.29 ± 0.35
50CTS/50TA + 1% Fe(III)	32.14 ± 0.71	9.89 ± 0.34	25.80 ± 0.15
80CTS/20TA	34.08 ± 0.96	6.12 ± 0.17	26.61 ± 0.79
80CTS/20TA + 10% Fe(III)	31.65 ± 0.78	5.16 ± 0.38	26.49 ± 0.39
80CTS/20TA + 5% Fe(III)	29.62 ± 0.87	4.47 ± 0.38	25.15 ± 0.49
80CTS/20TA + 1% Fe(III)	33.07 ± 0.48	7.03 ± 0.19	26.40 ± 0.29
20CTS/80TA	39.50 ± 0.34	6.91 ± 0.21	22.18 ± 0.42
20CTS/80TA + 10% Fe(III)	33.48 ± 0.37	5.72 ± 0.16	27.76 ± 0.21
20CTS/80TA + 5% Fe(III)	34.01 ± 0.44	2.62 ± 0.19	31.38 ± 0.24
20CTS/80TA + 1% Fe(III)	34.99 ± 0.37	5.72 ± 0.16	27.78 ± 0.21

The dispersive component was in the range of 22–31 mJ/m^2^ for all the samples, and the polar component was much lower, in the range of 2–9 mJ/m^2^. The complex formation with Fe(III) resulted in a decrease of surface free energy for each type of chitosan/tannic acid mixture compared to the material without Fe(III) addition. At the same time, the increase of Fe(III) amount resulted in the slight decrease of surface free energy. This was related to the cross-linking process between components which led to the conditionally bonding of the groups of tannic acid and chitosan. As a result, the less hydrophilic groups were free on the surface as they were blocked by Fe(III). Surface energy should below to improve the material integration with surrounding the interface. The hydrophilic surface is characterized by low surface free energy value which is beneficial for its application as a biomaterial film. It can be inferred from our analysis that materials composed 80CTS/20TA with 5%Fe(III) have the lowest surface free energy.

TA–Fe(III) complexes have been proposed as coatings due to the good adhering properties through covalent or non-covalent interactions (Fan et al. 2015). The surface free energy controls the cell–biomaterial interactions. The complexation of tannic acid by iron(III) results in lower surface free energy which creates a surface more attractive for surrounding and better integration. The hydrophilic surface has low surface free energy value which is beneficial in materials’ biomedical applications.

### Maximum tensile strength

**Fig. 3:**
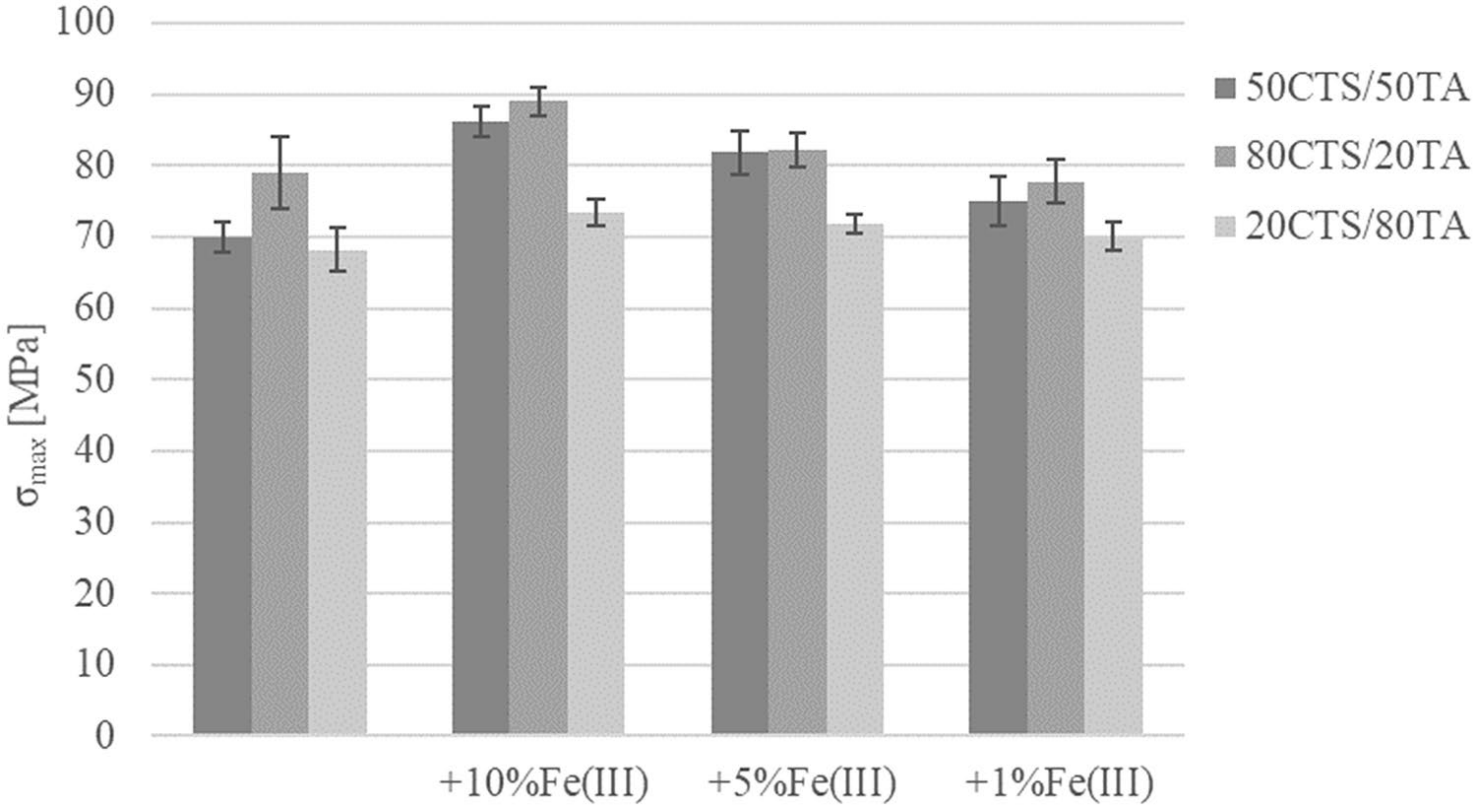
The maximum tensile strength of chitosan/tannic acid mixtures cross-linked by the Fe(III) ions in 1, 5, and 10% addition (*p < 0.05 vs. control)

The maximum tensile strength is an important parameter to consider a material application. Materials based on chitosan without any modifications may not find biomedical applications as they dissolve immediately in aqueous conditions. In our study, we mixed chitosan with tannic acid. Moreover, Fe(III) ions were added for the further improvement of material properties. As it may be observed in Fig. 3, the addition of Fe(III) ions improved the maximum tensile strength of films in composition 50CTS/50TA and 80CTS/20TA. This is related to the cross-linking process which occurred between components (Fig. 1). The obtained maximum tensile strength for materials with Fe(III) was higher than for materials without it. Thereby, it may be concluded that additional cross-linking of chitosan-based materials by metal complexation process led to improve the material mechanical properties.

### Blood compatibility

One of the most critical criteria to consider materials in blood–material contact for biomedical applications is the rate of hemolysis. The results of blood compatibility measurements are displayed in Table 2. Erythrocytes are sensitive to the hemolysis due to the shear stress (Weber et al. 2018).The rate of erythrocytes hemolysis decreased after the iron(III) addition; however for 10% Fe(III) content, it increased compared to 1 and 5%. Probably higher iron(III) content was high enough to be fully complexed with tannic acid and may lead to erythrocytes hemolysis. According to the ASTM F756-00 standard materials with a hemolytic index between 0 and 2% are classified as non-hemolytic, while materials with 2–5% are slight hemolytic and < 5% are classified as hemolytic (Pires et al. 2018). The hemolysis for chitosan/tannic acid materials is in the range 1.86–3.91 which classify them as slight hemolytic. Materials with Fe(III) have shown hemolysis below 2%, thereby categorizing them as non-hemolytic. Albeit, for all the proposed specimens, hemolytic index is below 5% implying that they are suitable for biomedical application.

**Table 2:** The rate of hemolysis for the samples based on chitosan and tannic acid–Fe(III) mixture

Specimen	Hemolysis rate [%]
50CTS/50TA	3.78
50CTS/50TA + 10% Fe(III)	0^a^
50CTS/50TA + 5% Fe(III)	0^a^
50CTS/50TA + 1% Fe(III)	0^a^
80CTS/20TA	1.86
80CTS/20TA + 10% Fe(III)	0.77
80CTS/20TA + 5% Fe(III)	0^a^
80CTS/20TA + 1% Fe(III)	0^a^
20CTS/80TA	3.91
20CTS/80TA + 10% Fe(III)	0.12
20CTS/80TA + 5% Fe(III)	0^a^
20CTS/80TA + 1% Fe(III)	0^a^

^a^Measured values for material were lower than for control

Blood is a mixture of numerous components. The majority of the fraction consists of erythrocytes. Chitosan-based materials are classified as non-hemolytic. Any modifications may not enhance the erythrocyte damage rate (Walczak et al. 2020). The complexation by Fe(III) in one side improves the surface properties and on the other side, it decreases the hemolysis rate. There is a lack of reported studies of tannic acid–Fe(III) complex-based materials on hemolysis rate. We proved that materials designed by us are safe to be applied as biomaterials.

### Platelet adhesion tests

**Fig. 4:**
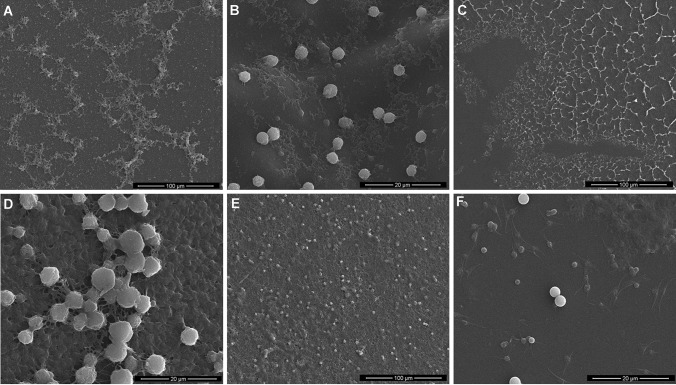
The morphology of the platelets adhered to the surface of **a** 50CTS/50TA, **b** 50CTS/50TA + 10% Fe(III), **c** 80CTS/20TA, **d** 80CTS/20TA + 10% Fe(III), **e** 20CTS/80TA, **f** 20CTS/80TA + 10% Fe(III) (Magnification 5000 × for **b**, **d**, **f**; 1000 × for **a**, **c**, **e**)

Protein adsorption is the first event that occurs when the surface of material comes into contact with blood. In the platelet adhesion studies, slightly fewer platelets were observed on the samples without Fe(III). Figure 4 shows that platelets with iron(III) are adhered to the surface of the film. Almost all platelets maintained a nearly round shape (A,D,F). For films based on 50CTS/50TA with 10% Fe(III) and 20CTS/TA with 10% Fe(III), no obvious pseudopodia-like structures were observed, implying a negative activation (Huo et al. 2018). However, for chitosan and tannic acid in ratio 80CTS/20TA complexed with 10% Fe(III), the pseudopodia-like structures were observed on the SEM images proving that a reduced tannic acid content can result in the undesirable platelets adhesion.

The reduction of platelet adhesion is a great challenge of modern biomedical science. It is well reported that tannic acid has excellent antiplatelet adhesion properties (Yang et al. 2017).The galloyl groups of TA are known to interact with proteins through hydrogen bonding, electrostatic and hydrophobic interactions. However, if it is mixed with chitosan, those interactions are already formed. Moreover, galloyl groups of TA were blocked through Fe(III) coordination what inhibits the fibrinogen by tannic acid. High chitosan content (80%) results in the presence of many hydrophilic groups which are not complexed by iron. They may interact with protein and the platelet adhesion with formed pseudopodia-like structures is noticed. Materials with antiplatelet surfaces may find future application as platelet-repellent blood-contacting biomaterials.

### Establishing cell cultures on the experimental films

**Fig. 5:**
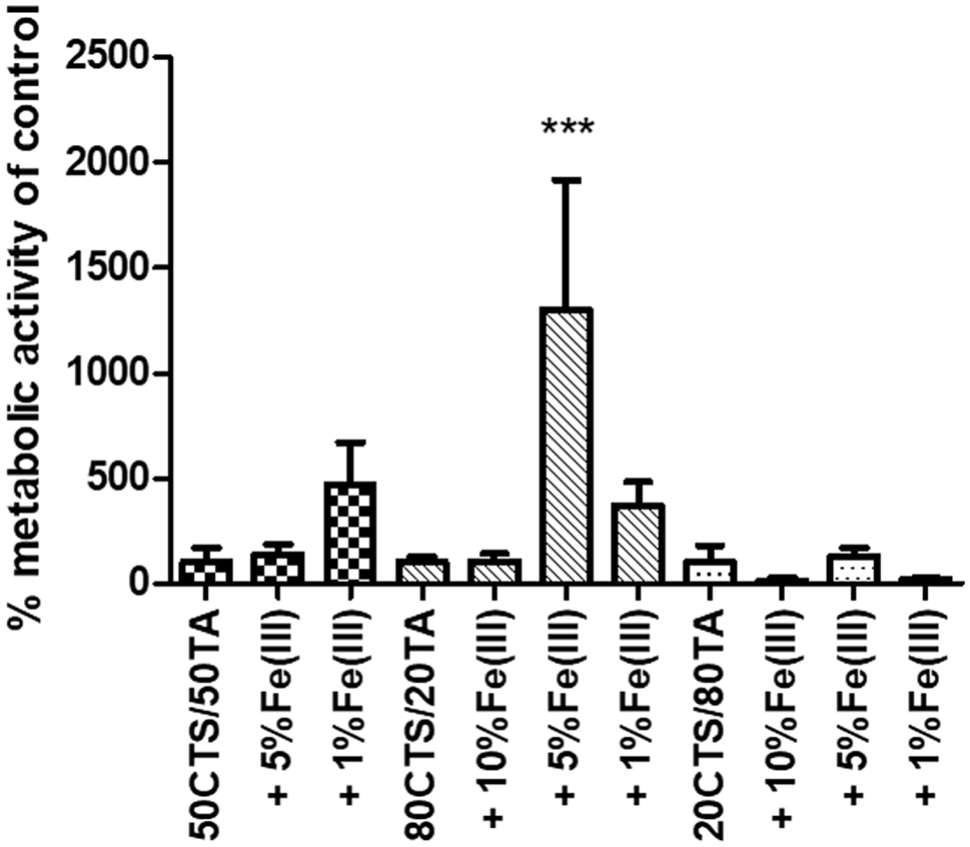
Metabolic activity of PDLSC on materials made out of different proportions of chitosan, tannic acid and Fe(III). Results presented as a percentage of viability on control materials (foils consisting only of 50CTS/50TA, 80CTS/20TA or 20CTS/80TA) assumed as 100%. ANOVA and post hoc Tukey used for statistical analysis; results statistically significant vs adequate control marked with*

Increased cell viability on materials made out of 80CTS/20TA + 5% Fe(III) was the only result of statistically significant versus control (Fig. 5). A tendency to higher viability contained cells grown on the materials consisting of 50CTS/50TA and 80CTS/20TA, both with the addition of 1% Fe(III). However, these results are not statistically significant. Visibly lower viability was observed in cells cultured on materials made out of 20CTS/80TA with the additions of 10% and 1% Fe(III), but these results are not statistically significant either. Therefore, it can be concluded that materials containing 50CTS/50TA and 80CTS/80TA and lower (5% and 1%) concentrations of Fe(III) support cell viability; whereas, materials containing 20CTS/80TA slightly impair cell viability. TA–iron(III) complexes have been reported to be able to adhere to various surfaces and increase their biocompatibility (Wang et al. 2017). Interesting studies were reported (Liu et al. 2018) where over 85% of cancer cells exposed on TA–Fe(III) complex were killed. This provides a green light for further material study in anticancer therapy.

## Conclusion

Chitosan and tannic acid mixture has been already tested for potential biomedical applications (Kaczmarek et al. 2019a). However, due to their low stability, it is imperative to modify them. In the present study, the complexation by iron(III) was proposed. Chitosan/tannic acid materials with Fe(III) showed good surface properties as low surface free energy. Moreover, they are found to be non-hemolytic and biocompatible for human cells. Thereby, it can be inferred that the metal complexation method is a good alternative to improve materials properties compared to costly cross-linking processes. The most optimum composition of tested material for the biomedical application was determined as 80CTS/20TA + 5%Fe(III). This material had appropriate physicochemical properties, was non-hemolytic and resulted in a significant increase of cell viability compared to other materials.

## Data Availability

The raw/processed data required to reproduce these findings cannot be shared at this time as the data also forms part of an ongoing study.
